# Hospital and Institutional News

**Published:** 1919-10-11

**Authors:** 


### HOSPITAL AND INSTITUTIONAL NEWS.

### MR. RALPH BANKES AND MILK ADULTERATION.

When dealing last week in The Hospital (October 4, 1919, p. 4) with the unsatisfactory state of the law on milk adulteration, and with the difficulty of indentifying and dealing with the true culprits in these cases, a phrase was used inadvertently from which it might be inferred that Mr. Ralph Bankes, the magistrate at the South-Western Police Court, is out of sympathy with the movement to ensure purity in our milk supplies. As a matter of fact, amply proved by his very urgent and common-sense letter in the *Times* of September 23 on this subject, Mr. Bankes is a strong supporter of the pure-milk crusade, and recognises as well as anyone the necessity for amendments in the law to secure that punishment for evasions shall fall on the right shoulders. We regret that, quite unintentionally, any words were used capable of a contrary construction.

### IMPROVEMENTS AT QUEEN CHARLOTTE'S.

Since the publicity given in The Hospital to the existent conditions within Queen Charlotte's Hospital, we are informed that a steady upward trend of improvement has taken place. Both in quantity and quality the food is better. Hot night meals with attendance are set and prepared in the dining-room for night nurses. These meals had hitherto been taken at the point of duty, sitting around on cots or bath edges—anywhere. Handleless crockery has been collected and replaced; window-ledges in bedrooms cleaned. A night portress attends the hall-door, hitherto attended by a pupil in conjunction with her ward duties. The day nurses are less frequently called up in the night to attend cases. Brass polish is distributed generally to the wards. Courtesy and politeness are more general in giving instructions. It is an interesting fact that the " Seven Pupil Nurses " are seven fully-trained nurses who have experience of other hospitals and of war service behind them. The pupils have been blamed for daring to criticise the hospital management.

### SIR E. T. COOK AND THE SEAMEN'S HOSPITAL.

An interesting reminiscence of the past is recalled by the death of Sir E. T. Cook, the eminent journalist, who passed away last week. He was the son of the late Mr. S. Kemball Cook, Secretary of the Seamen's Hospital Society from 1859 to 1874, and it was at the Secretary's house of the old " Dreadnought " Hospital at Greenwich that Sir E. T. Cook, and his equally distinguished brother, Sir Charles Cook, K.C.B., spent their early years. Mr. Kemball Cook was one of the first hospital secretaries to see the need of collaboration between various medical educational establishments. Nearly fifty years ago he assisted Major Wilkinson, of St. Mary's, in the compilation of the first hospital statistical tables that were ever published, and in many ways fostered the growing demand for reform in the voluntary charities. He was a pioneer and a hard worker in his day, and his distinguished sons followed in his steps and have both made a name for themselves in their own era.

### WOMEN DOCTORS.

Records show that there are now over 2,250 women studying to take medical qualifications in this country, and at the beginning of this session, although exact figures are not at hand, we know there are some hundreds more entering. At the moment, there are something over one thousand women doctors in the country, and by the end of the next five years' course, even allowing for a prevalence of failures, the number of women practising medicine will undoubtedly be doubled. It is true that the educational fees are not high, but the disadvantage remains that for five years a woman must keep herself from her own resources, a prohibitive condition to many during the present high cost of living. Also there seems no doubt that there must be by 1923, in which year alone 665 women are expected to qualify, considerable overcrowding in the ranks of women doctors. It is an outlook to be given serious consideration by the many war-workers and college girls who are now rallying to medicine. Success in the medical profession depends on many personal factors besides the ability to pass students' examinations brilliantly or otherwise and this probably applies even more than usual in the case of the woman doctor.

### A HOUSING MEASURE.

A short time ago it was announced that the Ministry of Health was prepared to expedite the provision of dwelling accommodation by sanctioning schemes for the conversion of large houses into working-class flats, and for the conversion of huts or hostels into temporary houses. Proposals have been made to extend also to provision of middle-class dwellings on similar principles, and in view of the small publicity given to this announcement we venture again to call attention to it. In many districts the conversion of large houses into flats will provide an appreciable amount of accommodation in the minimum time, and we already have the successful example of several local authorities who have transformed huts and hostels into temporary dwellings. The Ministry is fully prepared to receive and consider all proposals from local authorities for the use of State-owned buildings, and when approved these works will be regarded as part of the authorities' housing scheme under Section 1 of the Housing and Town Planning Act, and will have claim to financial assistance accordingly. This is only an emergency measure, and affords no excuse for delay in permanent building.

### FIRST MONTH OF PROHIBITION.

Taking some reports of the first month of prohibition in America, we read that in California the effect has been to cut the total number of arrests for all offences by two-thirds, while those for drunkenness have, in the larger cities, at least, fallen to almost nil. Chicago shows a decrease in all phases of crime except murder. Boston gives the same story, fewer arrests, less illness, and fewer accidental or suicidal deaths directly traceable to alcohol. New York reports a decrease of fifteen per cent in crimes of all kinds compared with July of 1918, but, on the other hand, an increased use of drugs is noted by the Health Department, and police authorities state that drugs as a substitute for liquor make the criminals uncommonly hard to catch!—an interesting point, perhaps worth more attention than a first glance may accord. But the working of *prohibition* must continue for some time longer before judgment can be passed from such figures alone. We saw equally good or better results from our own alcohol *restrictions* during the war period.

### DRUG INDUSTRY IN FRANCE.

In the fight with a renewed German pharmaceutical and chemical industry the various States concerned are very differently placed, and, although the French have been highly successful at preparing substitutes for a number of German-owned articles, yet we read in their press that fears as to the impossibility of breaking the old monopoly are entertained. They tell us that we ourselves and U.S.A. have already prepared vigorously to enter the lists, and that we have the all-important raw material, even though we do not possess the refinement of French production. Also in contrast with their own shortage of coal, war and blockade did not affect raw materials in Germany, with the result that the coal-tar synthetics, dyes, and perfumery can be placed by the Germans on the market without delay, especially on the French frontier, and at prices which defy competition. During the war no particular steps were taken to anticipate these *post-bellum* conditions, and it is apparently the old case of not mending the roof because of the rain.

### CHILD STUDY.

The Child-Study Society of London, founded with the object of scientific consideration of the mental and physical condition of children, and also of educational methods with a view to gaining greater insight into child nature and securing sympathetic and scientific methods of training the young, has issued the following programme of lectures and discussions, held at the Royal Sanitary Institute at 6 P.M.: October 9, "Biting Insects and Children," by A. E. Shipley, M.A., D.Sc., LL.D., F.R.S.; October 23, discussion on "The Literary Needs of the Child," opened by C. H. Barker; November 6, "How to Make 'English' Live in the Child-mind," by O. A. Minns, F.I.S.A.C., F.E.S.; November 20, "The Pre-School Child," by David Forsyth, M.D., D.S.C.; December 4, "Religion in Education," by the Rev. F. W. Cobb, D.D. Details of membership and attendance may be obtained from the Secretary, 90 Buckingham Palace Road, S.W. 1.

### A MEDICAL MUSEUM.

We learn that the Wellcome Historical Medical Museum, which contains a most interesting and valuable international collection illustrating the whole history of medicine and the allied sciences, will be reopened at 54a Wigmore Street, W., on Monday, October 13, 1919.

### HEALTH VISITORS' TRAINING.

The Board of Education announces that the draft, dated July 10, 1919, of the Regulations for the Training of Health Visitors has been published for the required period, and has now been confirmed by the Board without amendment. The draft now becomes "The Board of Education (Health Visitors' Training) Regulations, 1919," dated September 13, 1919, and copies can be purchased through any bookseller (price 1d.), or directly from H.M. Stationery Office, Imperial House, Kingsway, London, W.C. 2 (price by post 1½d.).

### SALES OF HOSPITAL EQUIPMENT.

Auctioneers disposing of the equipment of demobilised hospitals have had the good judgment lately to send catalogues to secretaries of voluntary hospitals. To the discriminating there have been many bargains to be picked up, but often the lots are too large to be handled by modest buyers. However, linen, blankets, and other stores can be carefully stowed away for future use. At a recent sale of the effects of the Third Northern General Hospital, Sheffield, local charities were well represented. Seven thousand white cotton sheets were offered; the new ones in lots of fifty fetched £14 per lot, and the used ones, in lots of twenty-five, from £6 to £6 15s. Bales of twenty-five new unpacked blankets fetched £23 10s. each, and three thousand counterpanes realised £4 to £9 per lot of twenty. Dusters were eagerly bought at £5 10s. for 140. The auctioneer remarked that he had sold many things in his time, but shrouds had never come his way before. The crowd evinced no enthusiasm in these gruesome articles, which went at a very low price.

### A CHILDREN'S HOSPITAL FOR CAPE TOWN.

The first and only children's hospital in the Union of South Africa is to be started shortly at Cape Town as a memorial to Second Lieutenant, the Hon. Dennis Sydney Buxton, Coldstream Guards, who was killed in Flanders on October 9, 1917. The scheme is to start a cottage hospital for children, containing twenty-two beds, bearing Mr. Buxton's name. His parents, Lord and Lady Buxton, have contributed £5,000 out of the £6,000 necessary to meet the estimated cost of an institution of this size. If a substantial amount in addition is raised by subscription, it will be devoted to the provision of a special wing for tuberculosis cases. Of the first twenty-two beds, fourteen will be for European and eight for coloured children. The land, equipment, and maintenance will be provided by the Cape Hospital Board. No details are yet published as to the locality in view. One may presume that it is likely to be one of the delightful and salubrious suburbs which stretch from Cape Town round the foot of the mountain in a continuous string of "garden cities."

### THE SHORTAGE OF MEDICINE BOTTLES AND ITS DANGERS.

It is common knowledge that at the present time there is a grave shortage of glass medicine bottles, and at a recent meeting of the Council of the Pharmaceutical Society the matter came up for discussion, with the view of impressing on the authorities the urgent necessity for an adequate supply being available for the coming winter. One member of the Council informed the meeting that in some parts of London, owing to the shortage, medicines were being sent out in "poison" bottles. If this is so—and we can hardly imagine any pharmacist at a hospital sanctioning the procedure—it constitutes a grave danger in that the whole subject of preventing the accidental administration of poisons by dispensing these in special bottles distinguishable by touch is defeated. In our opinion to send out medicines for internal use in a "poison" bottle is more deserving of censure than dispensing poisonous preparations in medicine bottles labelled "poison." Now that it is a legal obligation on the pharmacist to dispense poisonous preparations in bottles "rendered distinguishable by touch," it is to be hoped that action will be taken to prevent the misuse of these by utilising them for medicines intended for internal use.

### SIR COOPER PERRY ON CHILDREN'S HOSPITALS.

Sir Cooper Perry, of Guy's Hospital, has accepted an invitation by the Mayor of Bermondsey and the War Memorial Committee to assist in the establishment of a hospital for children as a war memorial. Sir Cooper Perry told the Committee that he can hardly imagine a better war memorial than a children's hospital. Strictly speaking, there are no children's wards in Guy's Hospital, and the children are put in the general wards. This is a matter that has caused a great deal of controversy. They have a small babies' ward, and are building an extension to contain fifty-four cots. When that is completed they will, of course, remove some of the children from the general wards into the cots, but obviously there is plenty of room for another children's hospital. In these days a children's hospital is almost invariably associated with a child-welfare scheme. In his opinion the children will become more and more the care of the State, and before long the State will take over the entire welfare of the children. Even when that is done, a voluntary children's hospital will still be useful. Such a place will more surely maintain efficiency, as it will respond to criticism in a way that no official place will. They are sure in Bermondsey to require a large out-patient department, but they will need some beds as well. He does not think he can give any formula as to the relation the out-patient department should bear to the in-patient, but twenty-five beds would make a very small place. A rather good size would be something like the Evelina Hospital for Sick Children, Southwark Bridge Road, which has sixty-five beds. According to the statistical returns of the London hospitals, the average cost for maintenance is £87 per year per occupied bed. He recently spoke to an architect on the subject, who estimated it would cost £100,000 to build a hospital to contain 100 beds. That is just about double what it would have cost before the war, but perhaps the figure quoted is a large one. It is best to commence with large administrative blocks, with just a few beds at first, as they can always build more wards, but they cannot pull the administrative block about once it is built. He offered to co-operate with the Mayor and the Medical Officer of Health for the County of London in drawing up a report for the consideration of the War Memorial Committee, and his offer was warmly accepted.

### A HOSPITAL SECRETARY AND THE STRIKE.

Lord Claud Hamilton, M.P., Chairman of the G.E.R., and Treasurer of the Railway Servants' Orphanage, has found his two positions incompatible in consequence of the railway strike. As chairman of a railway company he is inevitably entangled in the dispute; as treasurer of the railwaymen's orphanage he cannot but realise that the issue of an appeal at the moment is not likely to find a fair hearing from the public. If to secure that was his object, and time permitted, Lord Claud could have been content to suggest that the committee should temporarily withhold the appeal. But instead of this, or because time did not permit, he has announced that his signature must be withdrawn from it. This is very unfortunate for Lord Claud and for the orphanage of which he is treasurer. It is no use crying over spilt milk, but we cannot help saying that every step should have been taken to avoid this. For instance, the secretary of the orphanage ought to have kept in touch with the officials of the union, so as to hear from them whether the rails were clear (so to speak) for the issue of the appeal. Perhaps the secretary had reason to suppose that the appeal could be issued in front of the strike. If so, we can only sympathise with him in his present difficulty. But it shows, once again, that hospital secretaries must also be statesmen, for their work's success depends on an inside knowledge of public affairs.

### THE RED CROSS IN PEACE TIME.

One step towards the perpetuation of the close relation occasioned by the war between the public and the voluntary hospitals, between which the Red Cross and the Order of St. John acted as the bridge, has been taken. The Joint Council is to continue its work in peace, and both the Order and the Red Cross are equally identified with it. The future joint working of the two corporations has been determined by an agreement entered into on September 4, and since published. The agreement establishes a Joint Council of Control, which Council will consist of the Grand Prior of the Order, the Chairman of the Council of the Red Cross Society, and not more than forty-eight other members. We print the chief powers of the Joint Council on page 42.

### DEATH OF A HOSPITAL CHAIRMAN.

The Royal Victoria and West Hants Hospital, Bournemouth, and the Royal National Sanatorium have both lost their Chairman with the death of Mr. D. H. W. Robson-Burrows, an old inhabitant of Bournemouth, who died after six months of intermittent illness at the comparatively early age of fifty-two The Robsons, as the men in previous generations were known, have been associated with Bournemouth for several generations, and Mr. Robson-Burrows was for about seventeen years chairman of the Royal National Sanatorium. He had been chairman of the House Committee of the Royal Victoria Hospital before its amalgamation with the sister institution, and was elected to the chairmanship of the joint hospital in 1913. His wide experience of institutional management led to his becoming a member of the War Relief Committee, under which the treatment of wounded soldiers and sailors was arranged. Like most hospital men of ability, he was a man of many interests: sport, clubs, public objects of various kinds, found in him an active supporter. Golf, rowing (at Trinity Hall, Cambridge), football, and social clubs in the district formed his recreations, and he had a talent for managing voluntary organisations of every kind. He will be missed in many quarters, and Mr. Gordon M. Saul, the Secretary of the Royal Victoria and West Hants Hospital, must know as well as any man the quality of Mr. Robson-Burrows' work.

### NURSES' REGISTRATION IN AUSTRALIA.

The Nurses' Registration Bill, providing for the training, qualification, and registration of nurses, is at present before Parliament. A much objected-to provision is that, instead of a board of seven members representing various interests, including the medical profession, the Governor in Council may appoint five members of the public service as a board. This board may hold examinations of nurses or appoint examiners representing the medical profession, registered nurses, and public hospitals. The majority of the nursing profession is averse to a board of civil servants having any say in the matter of nurses' qualifications, and it is quite likely that the Bill will again be thrown out.

### SHOULD THE POLICE HAVE A HOSPITAL?

The Departmental Committee appointed to inquire into the medical service provided for the Metropolitan Police proposes, among other things, a hospital reserved for London policemen. The force numbers 20,000 men, for whom, at ten beds per thousand, 200 beds would be desirable. The Committee would be content, however, with a hospital with 150, at least at first. It will be noticed that though London has far fewer beds per thousand than experts desire, yet the proposed proportion of ten per thousand for the police shows that policemen run more risks of disease and accidents than the rest of us. Indeed, the proportion of beds suggested for them is about twice as great as even generous estimates for civilians used to ask. This lets a light into the nature of policemen's lives which, till lately, the public were content to take for granted. If the proposed hospital had its 150 beds in daily occupation, the cost is expected to be £25,000 a year on the basis of a weekly expense of £3 per patient. The whole annual charge would be £40,000, to meet which a levy of 6d. per week per man, and a proportionate sum for higher ranks, is proposed. The staff would include two resident medical officers, the senior of whom would receive a salary of £200 per annum and the junior £100. The visiting staff would also be paid.

### THE MASONIC HOSPITAL FOR PAYING PATIENTS.

The movement in favour of a hospital for paying patients of limited means which is being initiated by the Freemasons has already been referred to in The Hospital. The Lord Mayor is the Treasurer, and has arranged a meeting at the Mansion House on Friday, October 10, of the supporters of the scheme. The idea is to utilise one of the buildings in which the members of the Craft have been conducting their war work. They had three military hospitals, of which one was the Bishop of London's palace at Fulham, and another the building in the Fulham Road which was formerly the Chelsea Hospital for Women. The latter is to be the permanent Masonic Hospital, and will be used for members of the Craft of limited means and for their families. The building has been acquired and is equipped with modern appliances and an $x$-ray and electrical department. It will accommodate forty patients, and in respect of the charges the patients will be encouraged to pay a small fee to the surgeon for operations. Primarily it is intended to help that class of patient who otherwise would have to resort to the free wards of the voluntary hospitals. Because of the increased cost of hospital administration, the charges will be somewhat higher than originally contemplated, and for the present and until they can be reduced will probably be on the basis of £3 per patient per week. But in an ordinary three weeks' operation case a patient, we learn, should be able to secure treatment for an inclusive sum of about £15. The Freemasons are also contemplating the establishment of a Samaritan Fund to assist patients who cannot afford to pay even these small charges.

### THIS WEEK'S DRUG MARKET.

The settling of the railway strike has had a healthy effect in the drug and chemical markets, and business has improved considerably. Orders that had been delayed last week are now being placed, and when the congestion caused by the stoppage of train traffic has been removed we may expect to see a still larger volume of business. In fact, there is a much healthier feeling all round. Generally speaking, prices are well maintained. Menthol is again dearer, and the same applies to Japanese peppermint oil. Calomel, corrosive sublimate and other salts of mercury are rather lower in price as a result of a reduction in the price of quicksilver. Persian opium has been somewhat reduced in price. Cloves have again advanced in value. There is practically no change in the position of the synthetic drugs. Atropine is cheaper.

